# Crop Adaptation to Elevated CO_2_ and Temperature

**DOI:** 10.3390/plants11030453

**Published:** 2022-02-07

**Authors:** James Bunce

**Affiliations:** Adaptive Cropping Systems Laboratory, USDA-ARS, Beltsville, MD 20705-2350, USA; buncejames49@gmail.com

There is no ambiguity about the fact that both atmospheric CO_2_ levels and air temperatures are continuing to increase. It has only recently been recognized that the combination of these changes is likely to have a net negative impact on the production of many of our most important food crops. Therefore, concurrent with efforts to reduce emissions of CO_2_ and other gases which warm the atmosphere, efforts should be made to adapt crops to the conditions of elevated CO_2_ levels and temperature.

In response to the solicitation of articles on this topic, twelve articles have been published in this Special Issue of *Plants*, reflecting strong current research interest in this topic, as well as the diversity of relevant approaches.

Gavelienė et al. [1] tested the effects of warming on the root morphology of two species of lupine, one invasive and one noninvasive, and found that the two species had contrasting responses, which might affect their adaptation to climate warming.

Gardi et al. [2] examined growth and water use efficiency responses to elevated CO_2_ levels among 15 landrace and 15 released lines of barley from Ethiopia and found a large diversity of responses, suggesting that genetic improvement should be feasible in this important crop species.

Marcos-Barbero et al. [3] screened sixty bread wheat genotypes for grain yield at elevated CO_2_ and high-temperature conditions and found a large range of yields under those conditions, identifying genotypes that displayed promise of adaptation to climate change.

Jurkoniene et al. [4] examined the effects of warming on the IAA content and ethylene production of two lupine species with contrasting invasiveness and found more flexible responses in the invasive species.

Barickman et al. [5] compared the growth of basil at low, moderate, and high temperatures at ambient and elevated CO_2_ levels and found that elevated CO_2_ levels reduced photosynthesis at high temperatures but increased it at moderate and lower growth temperatures.

Ben Marium et al. [6] conducted a meta-analysis concerning the impacts of elevated CO_2_ levels, elevated temperature, and drought on the yield and grain quality of cereals and found that the beneficial yield responses to elevated CO_2_ levels were offset by both high temperatures and drought stress, with a general negative impact of elevated CO_2_ levels on nutritional quality.

Chen and Setter [7] examined the responses of tuber formation in potato to elevated temperature and CO_2_ levels and found that elevated CO_2_ levels partly compensated for the inhibition of tuber growth caused by elevated temperatures and that high temperatures at tuber initiation were especially important in this species.

Jayawardena et al. [8] examined the responses of nitrogen uptake and metabolism to elevated CO_2_ levels and temperature in tomato in great detail and found that the decreased nitrogen uptake and assimilation in response to the combined treatments probably resulted from decreased plant demand for nitrogen.

Bourgault et al. [9] tested the hypothesis that an elevated CO_2_ level only increases root growth in topsoil, not at depth. They conducted a detailed root-growth analysis in a FACE experiment with lentil, and found that in some cases, root growth at depth also increased at elevated CO_2_ levels.

Ma et al. [10] analyzed the response of sugar-metabolism-related genes to elevated CO_2_ level treatment in the goji berry to provide a molecular explanation of the reduced sugar content of these fruits when plants are grown at elevated CO_2_ levels.

Wang and Liu [11] provided a review of the effects of heat and elevated CO_2_ levels on the yield and grain quality of wheat, one of the crops in which negative effects of climate change on grain quality were first noticed.

Ziska [12] reviewed data concerning whether newer crop varieties are better adapted than older ones to high CO_2_ levels and suggested that examining the genetic responses of weedy relatives of crops to the changes in atmospheric CO_2_ that have recently occurred may provide a useful source of genetic traits, which could improve the responses of crops to future CO_2_ levels.

I hope that this compilation of research papers and reviews illustrates the broad range of relevant research on the topic of Crop Adaptation to Elevated CO_2_ and Temperature and stimulates additional research on this critical topic.

## Data Availability

Not applicable.
